# Supplementary material for: The Uncomfortable Truth: Open Thoracotomy versus Minimally Invasive Surgery in Lung Cancer: A Systematic Review and Meta-Analysis
Source: Cancers (Basel). 2023 May 5;15(9):2630. doi: 10.3390/cancers15092630 (PMC10177030; doi:10.3390/cancers15092630)

Figure S1. Forest plot of comparing age in study population according to surgical approach.

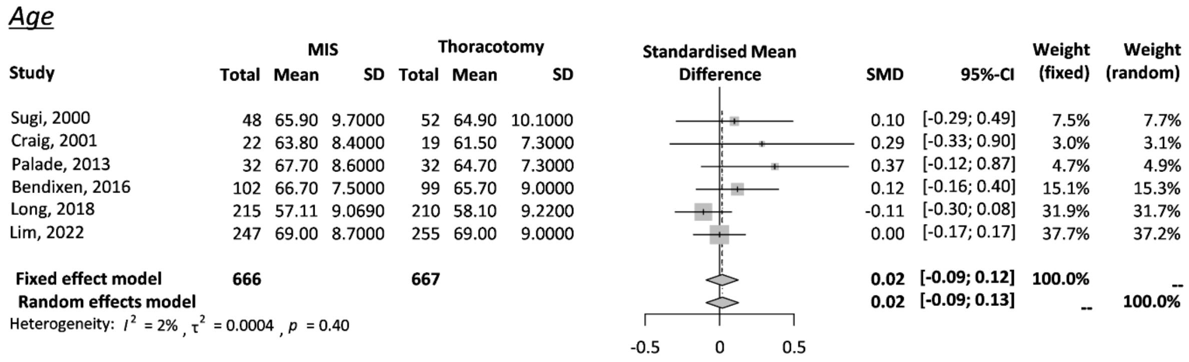

MIS, minimally invasive surgery

Figure S2. Forest plot of comparing male proportion in study population according to surgical approach.

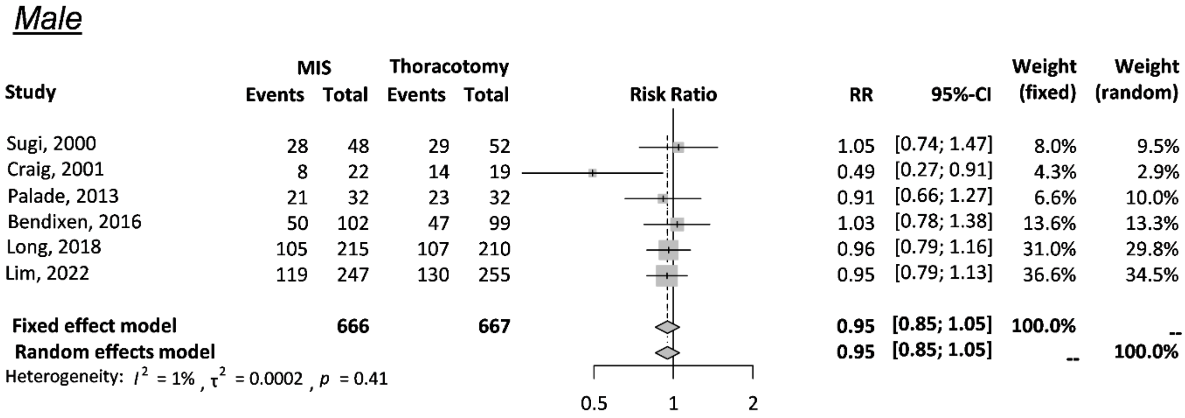

MIS, minimally invasive surgery

Figure S3. Forest plot of comparing adenocarcinoma proportion in study population according to surgical approach.

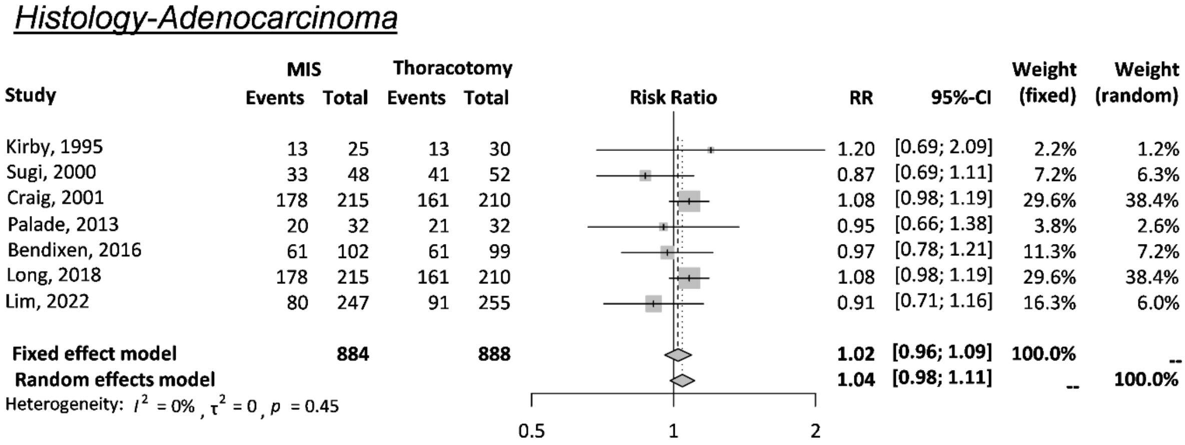

MIS, minimally invasive surgery

Figure S4. Forest plot of comparing tumor size in study population according to surgical approach.

Tumor size (with available data)

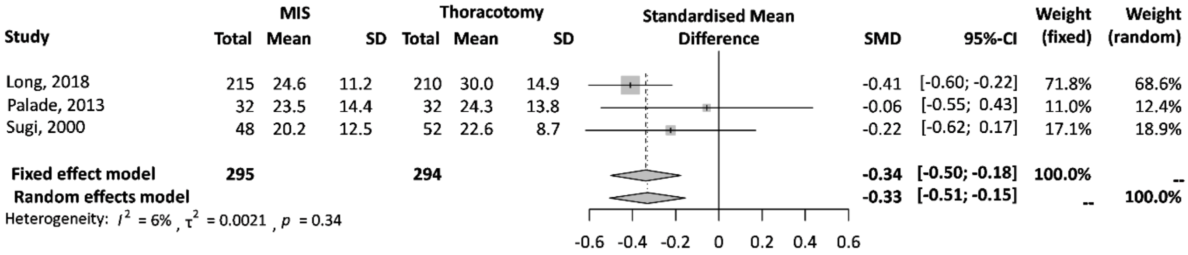

Figure S5. Forest plot of comparing proportion of patients with prolonged pain according to surgical approach.

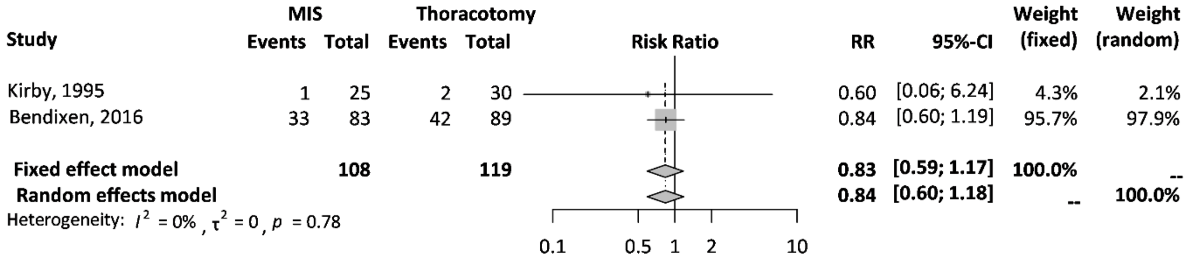

Supplement: Supplementary file 1 [file cancers-15-02630-s001.zip › cancers-2344321-supplementary.pdf]
